# Multiple implications of an active site phenylalanine in the catalysis of aryl-alcohol oxidase

**DOI:** 10.1038/s41598-018-26445-x

**Published:** 2018-05-25

**Authors:** Juan Carro, Pep Amengual-Rigo, Ferran Sancho, Milagros Medina, Victor Guallar, Patricia Ferreira, Angel T. Martínez

**Affiliations:** 1 Centro de Investigaciones Biológicas, CSIC, Ramiro de Maeztu 9, E-28040 Madrid, Spain; 2 Barcelona Supercomputing Center, Jordi Girona 31, E-08034 Barcelona, Spain; 3 Department of Biochemistry and Cellular and Molecular Biology, and BIFI, University of Zaragoza, E-50009 Zaragoza, Spain; 4 ICREA, Passeig Lluís Companys 23, E-08010 Barcelona, Spain

## Abstract

Aryl-alcohol oxidase (AAO) has demonstrated to be an enzyme with a bright future ahead due to its biotechnological potential in deracemisation of chiral compounds, production of bioplastic precursors and other reactions of interest. Expanding our understanding on the AAO reaction mechanisms, through the investigation of its structure-function relationships, is crucial for its exploitation as an industrial biocatalyst. In this regard, previous computational studies suggested an active role for AAO Phe397 at the active-site entrance. This residue is located in a loop that partially covers the access to the cofactor forming a bottleneck together with two other aromatic residues. Kinetic and affinity spectroscopic studies, complemented with computational simulations using the recently developed adaptive-PELE technology, reveal that the Phe397 residue is important for product release and to help the substrates attain a catalytically relevant position within the active-site cavity. Moreover, removal of aromaticity at the 397 position impairs the oxygen-reduction activity of the enzyme. Experimental and computational findings agree very well in the timing of product release from AAO, and the simulations help to understand the experimental results. This highlights the potential of adaptive-PELE to provide answers to the questions raised by the empirical results in the study of enzyme mechanisms.

## Introduction

Elucidation of structure-function relationships is of key importance in the study of enzyme catalysis. Unveiling the mechanisms that lie behind the properties of a biocatalyst paves the way to widen its biotechnological applicability by means of enzyme engineering^1^. In this regard, aryl-alcohol oxidase (AAO) is an enzyme of biotechnological interest from the glucose-methanol-choline oxidase/dehydrogenase (GMC) superfamily. AAO catalyzes the oxidation of primary benzyl alcohols into their aldehyde counterparts using atmospheric O_2_ as co-substrate and yielding H_2_O_2_ as co-product^2–4^. In this way, it has shown its potential for the production of flavours and aromas, deracemisation of alcohol mixtures and the synthesis of precursors for the manufacturing of renewable polyesters^5^.

The ecophysiological role of AAO would be the supply of H_2_O_2_ to either ligninolytic peroxidases —which it acts synergistically with— or to trigger Fenton reactions during natural decay of lignocellulosic materials^5,6^. H_2_O_2_ production by AAO involves the redox-cycling of *p-*anisaldehyde^7^, a metabolite of AAO-producing species^8,9^. Its natural role as H_2_O_2_ producer may be exploited for biotechnological purposes by the development of enzyme cascades in which AAO and H_2_O_2_-consuming enzymes (peroxidases and peroxygenases) act concertedly. In recent years, the repertoire of AAO substrates has been enlarged by the discovery of new molecules the enzyme can oxidise, such as 5-hydroxymethylfurfural, 5-methoxymethylfurfural and their partially oxidised derivatives^10,11^, and secondary benzylic alcohols. Regarding the latter substrates, AAO stereoselectivity and activity was improved by means of computer-guided rational design that facilitated a more appropriate positioning of benzylic alcohols in the active site thanks to the removal of the Phe501 side chain^12^. AAO presents other potential applications in lignocellulose transformation and production of flavours and aromas^5^. Altogether, this variety of bioconversions renders AAO an enzyme with a bright future in biocatalysis.

**Figure 1:**
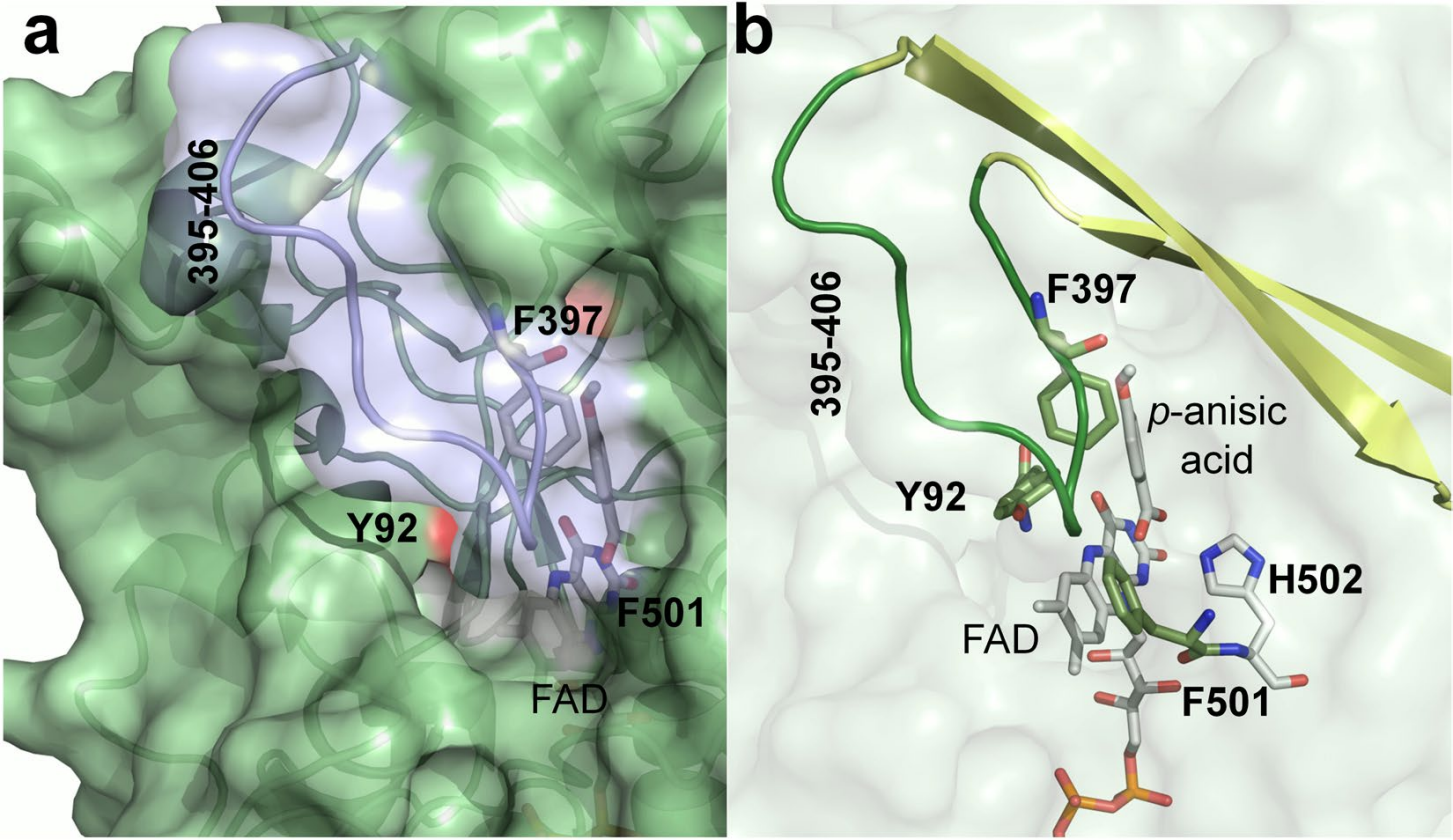
Surface loop and aromatic residues limiting access to the AAO FAD. (**a**) Area of channel opening on the AAO surface, with the 395–406 loop in transparent light blue. (**b**) Semitransparent surface showing the aromatic gate-keeping Tyr92, Phe397 and Phe501 aromatic residues (sticks in olive green), loop (green) and its contiguous β-strand residues (pale yellow cartoon), the catalytic His502 (grey) and FAD (grey), with one *p-*anisic acid molecule at the active site. From AAO:*p*-anisic acid structure (PDB **5OC1**)^17^.

The structure-function relationships of the model AAO from *Pleurotus eryngii* have been extensively investigated, and the roles of several residues important for catalysis have been elucidated^13–17^. The catalytic pocket of *P. eryngii* AAO, located near the flavin moiety of its FAD cofactor, is shielded from the outer environment by a triad of aromatic residues —Tyr92, Phe501 and Phe397— that form a hydrophobic bottleneck affecting alcohol and oxygen migrations into AAO active-site^18,19^. The roles of Tyr92 and Phe501 have been unveiled and are involved in: (i) establishing aromatic stacking interactions that guide the alcohol substrate to a catalytically competent configuration^13^; and (ii) compressing the active site to promote the reactivity with O_2_^14^, respectively. This residue is located in a loop characteristic of the AAO family (residues 395–406 in *P. eryngii* AAO), which partially covers the access to the active site^20^ (Fig. 1). Phe397 has been proposed to act as a barrier that prevents the free diffusion of molecules —substrates and/or products— in and out of the catalytic pocket protecting the flavin environment of AAO. Previous computational studies of substrate migrations into the active site —using the PELE algorithm^21^— suggested that Phe397 swung along with the alcohol substrate, helping it to reach the catalytic pocket^19^ (www.youtube.com/watch?v=CqSDn5OmagI). These results have encouraged the investigation of the role of Phe397 in AAO catalysis.

In this work, kinetic, ligand binding and diffusion studies on AAO and several mutated variants have been performed to enlighten our understanding on the role of Phe397 in catalysis. Moreover, molecular dynamics and new ligand diffusion computational studies using adaptive-PELE —a new version of the PELE algorithm that avoids the metastability of the ligands, thus saving number of processors and computation time^22^— have been employed to shed light on the catalytic implications of substituting Phe397 in AAO. Experimental findings and computational results agree very nicely and prove the applicability of the new adaptive-PELE to modelling the dynamic nature of protein-ligand interactions and its ability to unveil the mechanisms underlying binding in complex systems.

## Results

### Spectral properties and steady-state kinetics of AAO and its Phe397 variants

Wild-type recombinant AAO (hereinafter native AAO) and its F397A, F397L, F397Y and F397W variants were purified as holoproteins, after *Escherichia coli* expression and *in vitro* activation. The UV-visible spectra of all variants showed the typical flavin bands around 460 and 385 nm and shoulder around 500 nm (data not shown), indicating proper folding around the cofactor. 280 nm/460 nm absorbance ratios around 10–11 showed that the FAD cofactor was in the oxidized state and correctly incorporated into the protein for all variants.

**Figure 2:**
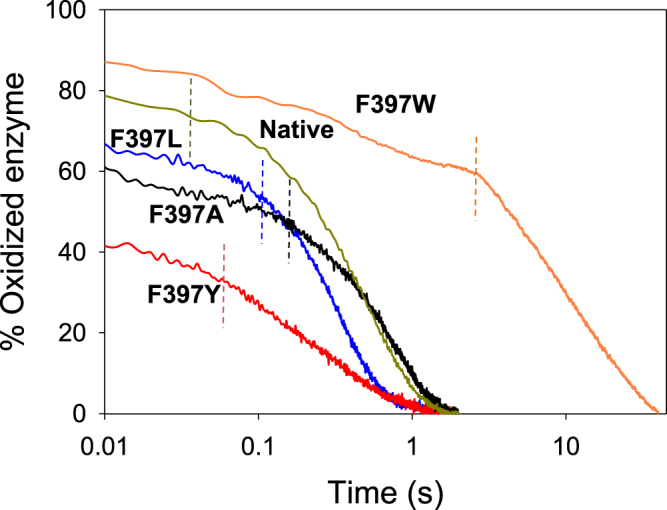
Redox state during turnover of native AAO and its Phe397 variants. Native (*green*), F397W (*orange*), F397L (*blue*), F397A (*black*) and F397Y (*red*) Native protein and its variants (~10 µM) were mixed with an excess of *p*-methoxybenzyl alcohol in 50 mM sodium phosphate pH 6.0 at 25 °C under aerobic conditions. Lines show the time course of absorbance changes at the maxima of the flavin band I (in the 459 and 463 nm range, depending on the variant). Dashed lines separate the turnover phase from the drop in absorbance due to the depletion of O_2_.

The redox state of the cofactor during turnover was investigated by following the spectra of the AAO variants during oxidation of *p*-methoxybenzyl alcohol (initially saturated with air) until the enzyme was completely reduced. In the cases of native AAO and the majority of the variants —F397W, F397A and F397L— the most abundant species during turnover (initial reaction phase) is the oxidized enzyme, whereas the reduced species is the predominant for F397Y (Fig. 2).

Bi-substrate steady-state kinetics —measured as *p*-anisaldehyde production from *p*-methoxybenzyl alcohol at different O_2_ concentrations— revealed remarkable differences among native AAO and its F397 variants (Table 1). First, in all four variants, contrary to the native AAO^16^, the kinetics best fitted equation (1) describing a ping-pong mechanism, as revealed by the Hanes-Woolf plots of its bi-substrate kinetics (Supplementary Fig. S1). Then, all variants, with the exception of F397L, showed 2–3 fold lower turnover rates (*k*_cat_ values) than the native enzyme. Regarding affinity for the alcohol substrate, the F397Y variant showed the same Michaelis constant (*K*_m(Al)_) as the native protein, while the F397A, F397L and F397W substitutions increased *K*_m(Al)_ by ~2-, 9- and 11-fold, respectively. Consequently, the F397Y, F397A, F397L and F397W variants were ~3-, 4-, 10- and 21-fold less efficient (*k*_cat_/*K*_m_) oxidizing the alcohol substrate compared to the native AAO. Regarding the affinity for O_2_, all the variants, with the exception of F397A, showed increased affinity (lower *K*_m(ox)_) with regard to the native protein. Thus, F397Y, F397L and F397W were 1.4-, 1.6-, and 2-fold more efficient using O_2_ as an electron acceptor than the native protein. Finally, steady-state constants calculated from anisaldehyde release (as in previous experiments) were compared with those obtained from H_2_O_2_ release (in additional kinetic analyses under atmospheric O_2_ saturation). In all variants, *k*_cat_ and *K*_m(Al)_ tended to be almost identical using both approaches (Supplementary Table S1), as it had been previously reported for native AAO^23^.

**Table 1:** Steady-state kinetic constants of native AAO and its Phe397 variants.

	***k***_**cat**_ (s^−1^)	***K***_**m(Al)**_ (µM)	***k***_**cat**_ **/** ***K***_**m (Al)**_ (s^−1^·mM^−1^)	***K***_**m(ox)**_ (µM)	***k***_**cat**_ **/** ***K***_**m(ox)**_ (s^−1^·mM^−1^)
AAO^1^	129 ± 5	25 ± 3	5160 ± 650	348 ± 36	371 ± 41
F397Y	48 ± 1	25 ± 1	1920 ± 65	94 ± 4	512 ± 20
F397W	68 ± 1	280 ± 8	240 ± 5	90 ± 4	718 ± 10
F397A	66 ± 1	54 ± 1	1224 ± 32	500 ± 10	133 ± 3
F397L	115 ± 1	226 ± 4	506 ± 10	190 ± 4	610 ± 13

The constants for *p*-methoxybenzyl alcohol (Al) and O_2_ (Ox) were measured as the *p*-anisaldehyde produced in bi-substrate kinetic experiments, performed in 50 mM sodium phosphate (pH 6.0) at 12 °C. ^1^From Ferreira *et al*.^13^. Means and standard deviations estimated from the fit to equation (1). All kinetics were measured by triplicates.

### Rapid kinetics of the two half-reactions for the Phe397 variants

**Figure 3:**
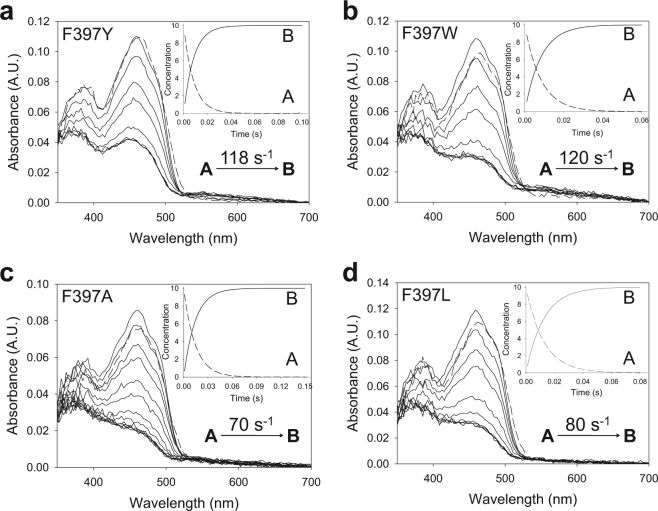
Time course of reduction of Phe397 variants with *p*-methoxybenzyl alcohol. (**a**) Spectra of F397Y mixed with 600 μM substrate measured at 0.003, 0.005, 0.01, 0.015, 0.02, 0.025, 0.06 and 0.1 s after mixing. (**b**) Spectra of F397W mixed with 2400 μM substrate recorded at 0.003, 0.01, 0.02, 0.03, 0.05, 0.07, 0.2 and 0.5 s. (**c**) F397A reduction spectra with 1200 μM substrate at 0.003, 0.01, 0.02, 0.03, 0.04, 0.05, 0.06, 0.08 and 0.3. (**d**) Spectra of F397L mixed with 2400 μM substrate at 0.003, 0.01, 0.02, 0.03, 0.05, 0.07, 0.1, 0.13 and 0.4 s. Enzyme (~10 µM) reactions were performed in 50 mM sodium phosphate, pH 6.0, at 12 °C. Dashed lines correspond to the oxidized enzymes before mixing. Data were globally fitted to a single-step model described from initial species A to final species B (shown in insets). Estimated *k*_obs_ is represented in each panel.

In the light of the above results, the reductive and oxidative half-reactions of the F397 variants were analyzed to unveil the rate-limiting step during catalysis. The spectra collected during the reductive half-reactions of all the variants indicated an essentially irreversible two-electron reduction of the flavin, in agreement with the previously reported hydride transfer reaction for the native AAO^16^. Global analyses of the spectral evolution were fitted to a one-step model (A → B) in all cases (Fig. 3). The values of the observed rate constants (*k*_obs_) at different substrate concentrations exhibited a hyperbolic dependence on the alcohol concentration (Supplementary Fig. S2) that allowed the determination of the reduction rate constant (*k*_red_) and the dissociation constant (*K*_d(Al)_) upon fitting to equation (3). For native AAO (spectral changes not shown in Fig. 3) and the F397A and F397L variants, the *k*_red_ values (Table 2) were of the same range of the previously determined *k*_cat_ values (Table 1) indicating that the reductive half-reaction is the rate-limiting step in catalysis. Nevertheless, variants F397Y and F397W showed *k*_red_ values 3- and 2-fold higher than the respective turnover rates, suggesting that reductive half-reaction is not the limiting step in these variants. For all the F397 variants, *K*_d(Al)_ values were similar to the *K*_m(Al)_ estimated under steady-state conditions.

**Table 2:** Transient-state kinetic constants for the reductive and oxidative half-reactions of AAO and its Phe397 variants.

	***k***_**red**_ (s^−1^)	***K***_**d(Al))**_ (µM)	***k***_**red**_ **/** ***K***_**d**_ (s^−1^·mM^−1^)	^**app**^ ***k***_**ox1**_ (s^−1^·mM^−1^)
AAO	115 ± 3	31 ± 2	3710 ± 258	770 ± 40
F397Y	150 ± 3	41 ± 3	3660 ± 277	770 ± 70
F397W^1^	124 ± 3	292 ± 17	425 ± 10	689 ± 92
F397A	69 ± 1	61 ± 2	1130 ± 40	78 ± 4
F397L	87 ± 1	180 ± 7	483 ± 20	340 ± 10

The constants were measured using stopped-flow rapid spectrophotometry in 50 mM sodium phosphate (pH 6.0) at 12 °C under anaerobic conditions. ^1^The F397W constants for the first phase of the oxidative half-reaction show a hyperbolic dependence on O_2_ concentration (in contrast to the other variants) with *k*_ox_/*K*_d(ox)_ and *k*_ox_ values of 689 ± 92 mM^−1^s^−1^ and 156 ± 12 s^−1^ respectively, estimated from fit to equation (5). Means and standard deviations estimated from the fits to equations (3), (4) and (5). All kinetics were measured by triplicates.

**Figure 4:**
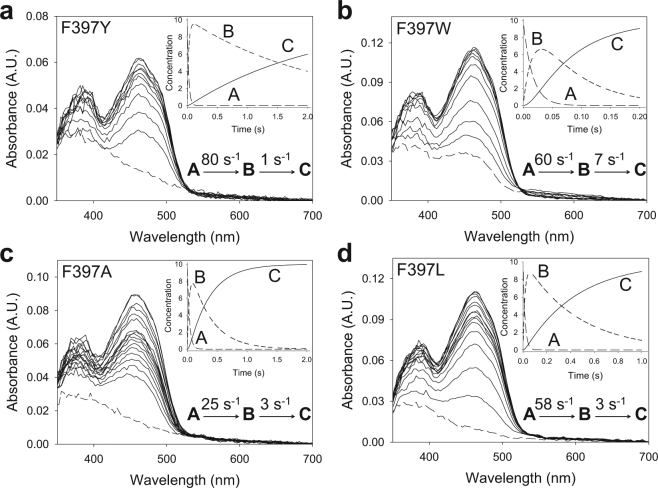
Time course of reoxidation of Phe397 variants with O_2_. (**a**) F397Y spectra measured at 0.002, 0.01, 0.02, 0.03, 0.04, 0.05, 0.06, 0.07, 0.08, 0.5, 1 and 1.5 s after mixing. (**b**) F397W recorded at 0.001, 0.005, 0.01, 0.02, 0.03, 0.04, 0.05, 0.06, 0.07, 0.08, 0.09, 0.1, 0.18 and 0.2 s. (**c**) F397A recorded at 0.002, 0.01, 0.02, 0.03, 0.04, 0.05, 0.06, 0.07, 0.08, 0.09, 0.5, 1, and 1.5 s. (**d**) F397L recorded at 0.002, 0.01, 0.02, 0.03, 0.04, 0.05, 0.06, 0.07, 0.08, 0.09, 0.1, 0.3, 0.5, 0.7, 0.9 and 1 s. Enzyme (~10 µM) reactions with O_2_ (136 μM) were performed in 50 mM sodium phosphate, pH 6.0, at 12 °C. Dashed lines correspond to the reduced enzymes. Insets show the evolution of species A, B and C after data fitting to a two-step process. The estimated *k*_obs_ for each phase is represented in each panel.

The oxidative half-reactions of the four Phe397 variants, and native AAO (not shown), fitted two-step model equations (A → B → C) describing a biphasic pattern (Fig. 4) where the first phase accounts for more than 70–80% of the total amplitude. For the F397Y, F397L and F397A variants, this *k*_obsA→B_ was linearly dependent on O_2_ concentration, allowing the determination of a second-order rate constant (^app^*k*_ox_) that was similar, 2- or 10-fold slower than that of native AAO, respectively (Table 2). For the F397L and F397A variants, a reverse rate constant was observed, *k*_rev_ ~15 s^−1^, which corresponds to the intercept of the y-axis (Supplementary Fig. S3A). On the contrary, the *k*_obsA→B_ for F397W showed a hyperbolic dependence on O_2_ concentration that fitted equation (5) and describes an encounter complex of a reduced flavin enzyme with O_2_ followed by flavin reoxidation (Supplementary Fig. S3B). The estimated transient-state second order rate constant, *k*_ox_/*K*_d(ox)_, ~689 s^−1^mM^−1^ (Table 2 footnote) agreed with the catalytic efficiency determined under steady-state conditions (Table 1). The second phase for all variants was too slow to be relevant for catalysis (*k*_obs2_ < 7 s^−1^), although it was independent of O_2_ concentration for the F397Y and F397W variants and dependent on it in the case of the F397L and F397A variants (Supplementary Fig. S3A, inset). The O_2_-independent slow phase in native AAO reoxidation had been previously attributed to the presence of damaged protein ensuing the stopped-flow experiments^24^.

### Studies on AAO:*p*-anisic acid complex formation and dissociation

**Figure 5:**
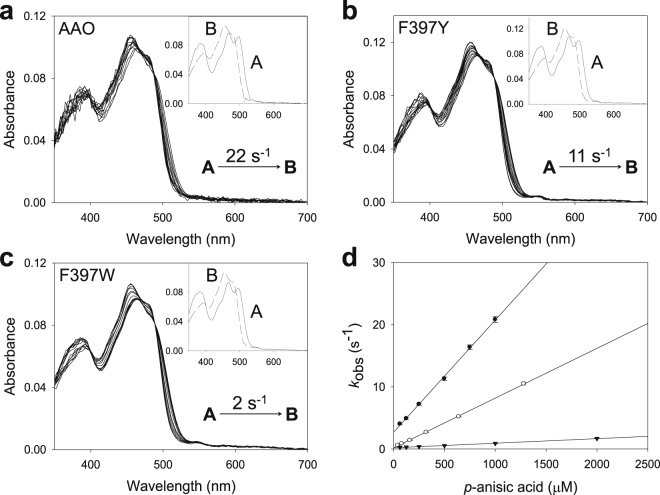
Spectral changes during formation of AAO:*p*-anisic acid complex. (**a**) Native AAO, mixed with 0.5 mM of the ligand at 0.003, 0.01, 0.02, 0.03, 0.04, 0.05, 0.065, 0.085, 0.1, 0.13, 0.15, 0.2 and 0.4 s. (**b**) F397Y, mixed with 0.65 mM of *p*-anisic acid measured after 0.005, 0.05, 0.1, 0.2, 0.3, 0.4, 0.5, 0.6, 0.7, 0.8, 0.9, 1, 2, 3 and 4 s, and (**c**) F397W mixed with *p*-anisic acid 1 mM recorded at 0.005, 0.02, 0.04, 0.05, 0.07, 0.1, 0.2, 0.4, 0.5, 0.7, 1, 2 and 5 s. Insets show disappearance of initial species A and formation of final species B. The rate constant obtained from the global fitting of the spectral changes is shown. (**d**) Fits of the obtained *k*_obs_ for each variant and ligand concentration to equation (6). Native AAO (filled circles), and F397Y (open circles) and F397W (inverted triangles) variants.

The differences between the *k*_cat_ and *k*_red_ values for both F397Y and F397W encouraged us to investigate whether product release has an effect on turnover. The study could not be carried out with *p*-anisaldehyde because the formation of the enzyme-aldehyde complex is too rapid, which prevents its detection by the stopped flow equipment. Therefore, this study (Fig. 5) was performed with the final product, *p*-anisic acid, whose crystallographic complex with AAO was recently solved (PDB **5OC1**)^17^. The rate constants for complex formation (*k*_for_) and dissociation (*k*_dis_) of native AAO and the F397Y and F397W variants are shown in Table 3. The *k*_for_ for F397Y and F397W were 2-fold and 2 orders of magnitude lower than that of the native enzyme, respectively. Differences in *k*_dis_ were also observed, since the *k*_dis_ values for these variants were at least 10-fold slower than that of the native AAO. The fast diffusion of the ligand in and out the active site of the F397L and F397A variants most likely impeded estimation of these parameters. The *K*_d(Ac)_ values calculated from the corresponding *k*_dis/_*k*_for_ ratios for native AAO and the F397Y and F397W variants (150, 31 and 271 µM respectively) agree well with the values obtained by differential spectrophotometry (Supplementary Fig. S4 and Table 3). F397Y showed the smallest *K*_d(Ac)_, which means that it is the variant that more tightly binds *p*-anisic acid. On the contrary, the *K*_d(Ac)_ for the F397L and F397A variants could not be estimated by differential spectrophotometry since saturation could not be attained in agreement with the above results.

**Table 3:** Dissociation constants, and formation/dissociation rates of *p*-anisic acid complexes.

	***K***_**d(Ac)**_ (µM)	***k***_**for**_ (s^−1^·mM^−1^)	***k***_**dis**_ (s^−1^)
AAO	170 ± 5	18.0 ± 0.3	2.7 ± 0.17
F397Y	25 ± 2	7.7 ± 0.1	0.24 ± 0.04
F397W	200 ± 12	0.70 ± 0.01	0.19 ± 0.01
F397A	nd	nd	nd
F397L	nd	nd	nd

The dissociation constants (*K*_d(Ac)_) of *p*-anisic acid complexes with AAO and its Phe397 variants were measured by differential spectroscopy, while the complex formation (*k*_for_) and dissociation (*k*_dis_) rates were measured by transient-state kinetics spectroscopy. All experiments were performed in 50 mM sodium phosphate (pH 6.0) at 12 °C. nd, not determined (too fast complex formation and dissociation). Means and standard deviations from the fit to equations (7) or (6). All data were measured by triplicates.

### AAO molecular dynamics and ligand diffusion simulations

MD simulations were carried out to study the dynamical behaviour of the five protein systems (native enzyme and its Phe397 variants). Loops are the secondary structures that most likely adopt different conformational structures. Hence, the motions of the loops Gln395-Thr406 and Ser89-Met95, where two of the main gate residues —Phe397 and Tyr92— are located, were investigated. However, the root-mean-square-deviation values obtained, as a function of time, were low (data not shown) indicating that there are only moderate displacements of these secondary structures along the dynamics of the protein.

Despite the small loop movements, a closer look at the distances between the gate residues indicates that the F397Y and F397W substitutions induce significantly shorter values (Supplementary Fig. S5A). Moreover, inspection of the protein structures shows that the side chains of the F397Y and F397W variants establish hydrogen bonds with Tyr92 with a frequency of 20% and 5%, respectively (Supplementary Fig. S5B), while the aliphatic variants and the native enzyme cannot develop such hydrogen bonds resulting in larger distances between gate residues. Altogether, the MD data suggests local constraints induced by the tyrosine and tryptophan substitutions in the gate region.

**Figure 6:**
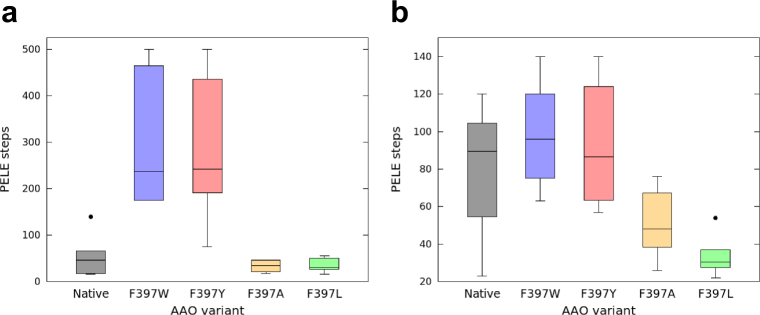
Boxplots of PELE steps required for ligand release from AAO and its Phe397 variants. (**a**) *p*-Anisic acid. (**b**) *p*-Anisaldehyde. Number of PELE steps is directly related to time. Grey, native AAO; blue, F397W; red, F397Y; yellow, F397A; and light green, F397L variants. Boxes contain 50% of the results (two quartiles), horizontal lines inside them indicate the mean value; while the upper and lower whiskers (vertical lines) contain the remaining quartiles (50%). The overall height of boxes (whiskers included) is indicative of the spread of the results. Isolated points represent results significantly different from the rest of data values.

The diffusion of products —*p*-anisaldehyde and *p*-anisic acid— and reactants —*p*-methoxybenzyl alcohol and O_2_— was also investigated with adaptive-PELE to explain the changes in kinetics observed for the mutants. The trajectory analysis of the acidic product in the aromatic variants clearly indicates a stabilization of the local minima at the gate, before ligand release, which does not take place during release of the aldehyde products. This energy minimum involves interaction between the carboxylic group and three amino acids, Ser393, Gln395 and Ser411, placed inside the catalytic cavity. The aromatic variants required in average ~3-fold and ~7-fold more simulation steps to release the aldehyde and acid, respectively, than the aliphatic ones (Fig. 6). Moreover, the number of PELE steps required to release both products also tends to be higher in the case of the aromatic mutants than for the native enzyme, especially for anisic acid due to the interactions of the carboxylic group with the three residues mentioned above.

**Figure 7:**
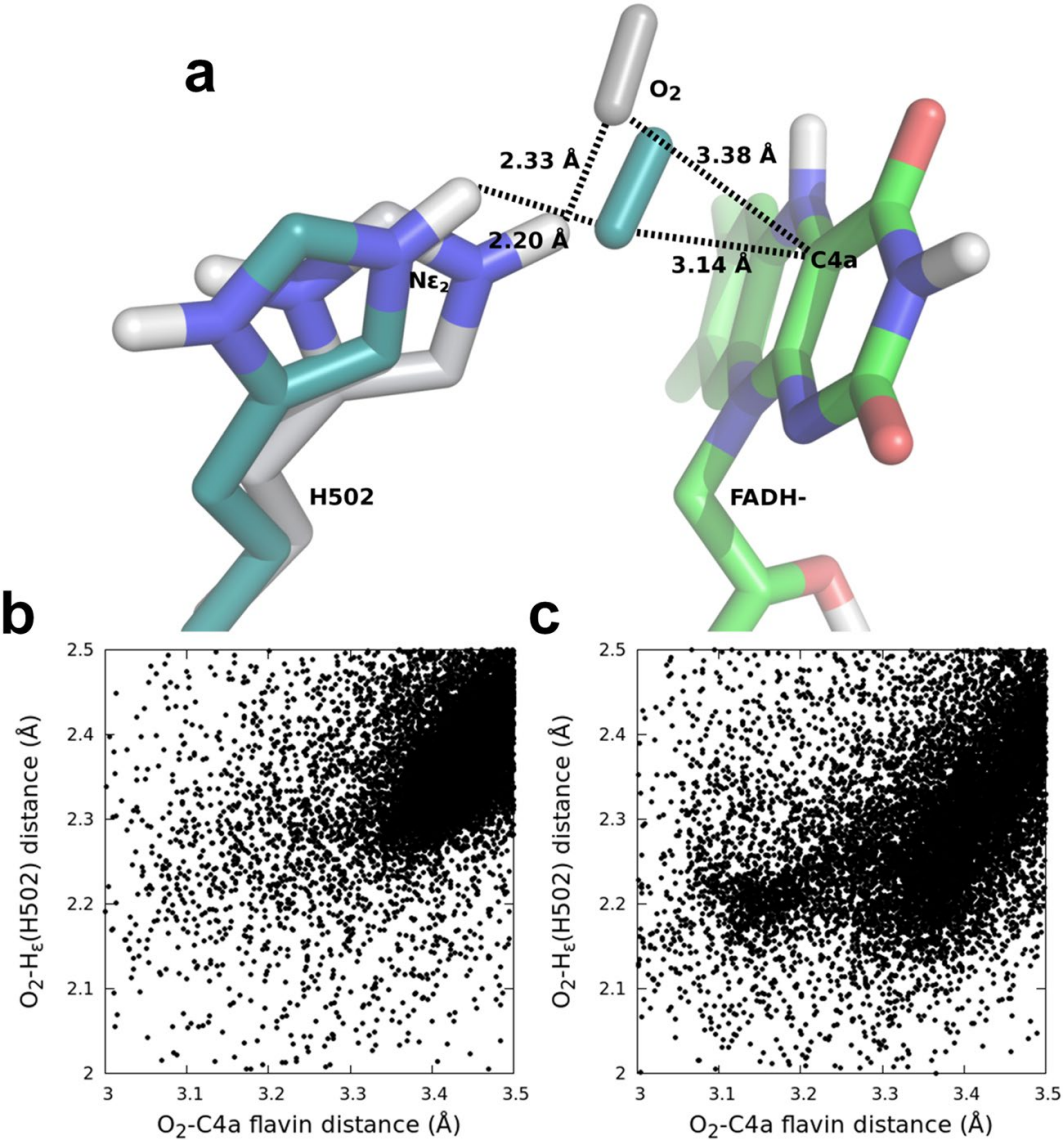
O_2_ diffusion in native AAO and its F397W variant. (**a**) Native and F397W final catalytic poses (CPK-colored molecules with grey and cyan carbon atoms, respectively) showing catalytic distances (in Å) to FAD C4a and His502. (**b** and **c**) Frequency of O_2_ poses as a function of the distances from O_2_ to His502 and C4a of native and the F397W variant, respectively.

Furthermore, the distances among any of the atoms of the O_2_ substrate and both the C4a locus of reduced FAD and the Nε_2_ of His502 (acting as a catalytic base in the reductive half reaction) were calculated for all the variants. Results suggest that the catalytic distances are similar in the native enzyme and variants, except for F397W that shows higher population at favourable —that is, shorter— distances (Fig. 7).

Finally, the energetic profiles of the *p*-methoxybenzyl alcohol catalytic distances (hydroxyl-to-His502 and pro*R-*H-to-flavin) in all protein systems were studied, as a measure of the binding effectiveness of the alcohol in the active centre. The energy landscape shows an additional energetic favourable region only for the F397W. In this new minimum, the side chain of the tryptophan interacts via hydrogen bond to the hydroxyl group of the alcohol substrate, resulting in an inefficient catalytic pose (Supplementary Fig. S6). This is in agreement with the experimental values, where the *K*_m(Al)_ and *K*_d(Al)_ are almost 11-fold higher for the F397W variant compared to the native protein.

## Discussion

Phe397 is situated at a loop characteristic of the AAO family, in a region prone to harbouring insertions and deletions amongst the members of the GMC superfamily. Representatives of this superfamily are monomeric or multimeric proteins, with the entrance to their active site partially covered by the adjacent monomer in the second case. Given their monomeric nature, AAOs and a few other GMCs have developed loop structures to control diffusion of molecules into their active sites. Among them, choline oxidase^25^, cholesterol oxidase^26^ and cellobiose dehydrogenase^27^ are notable examples, whose insertions are even longer than the AAO insertion. The latter insertion partially forms the 395–406 loop in *P. eryngii* AAO and encloses the active-site cavity from the outer environment (Fig. 1)^18^. Previous computational studies hinted that Phe397 oscillates with the substrate as a mechanism of gating^19^, similarly to what has been reported for phenylalanine residues in P450 enzymes^28^. A homologous phenylalanine residue is conserved in 50% of all the putative AAO sequences from basidiomycete genomes available at JGI (https://jgi.doe.gov). The multiple roles of this phenylalanine residue in catalysis, as revealed by studies on the *P. eryngii* model AAO, are discussed below.

Phe397 favours the correct positioning of the alcohol and its oxidation at the active site, as evidenced by the steep decrease of catalytic efficiency in the mutated variants. Such effect is most noticeable in F397W, which shows the lowest *k*_cat_/*K*_m(Al)_ value due to: i) the constrained distance between gate residues, and ii) the formation of a H bond with its reactive hydroxyl group that disrupts the substrate’s catalytic poses. The introduction of a bulkier residue at the Phe501 and Tyr92 positions of AAO also produced similar steric hindrances for catalysis^13,14,17^. Remarkably, the drop of the catalytic efficiency in F397Y is due to its small turnover rates, as affinity for alcohol (*K*_m(Al)_ and *K*_d(Al)_) remains invariable in this case. Furthermore, the reduction rates being higher than turnover in F397Y and F397W, indicates that, unlike native enzyme, the reductive half-reaction is not the rate-determining step in these two variants, as discussed below.

Phe397 also plays a role in flavin reoxidation by either compressing the active site to reduce the free diffusion of O_2_, or altering its redox environment. Evidence comes from the low oxidation rates estimated for the aliphatic mutants, in contrast to the ones obtained for the aromatic variants and the native AAO, which possess bulkier residues that constrain the catalytic pocket. However, the oxidative half-reaction does not limit the catalytic cycle in the aliphatic variants, as *k*_cat_ and *k*_red_ values are similar and the constants measured as H_2_O_2_ release are identical as those measured by aldehyde formation. Alteration of the FAD environment and electrochemistry might be involved in the decreased efficiency for oxidation of the F397A cofactor. Regarding the F397Y and F397W variants, they show high ^app^*k*_ox_, similar to the native protein. In the case of F397W variant, the obtained *k*_ox_ and *k*_red_ are similar indicating that the reductive and oxidative half-reactions are almost balanced, which agrees with its redox state during turnover. Computational simulations of F397W reoxidation revealed that withdrawal of His502 due to the presence of the tryptophan increases the ability of this variant for properly positioning the oxygen molecule during flavin reoxidation, bringing O_2_ and C4a closer than in the native enzyme (Fig. 7). However, the introduction of this bulkier residue could be hindering the oxygen diffusion into the active-site^19^ and thus causing the unusual saturation behaviour observed for this variant with increasing oxygen concentration. This saturation profile has been observed in cholesterol oxidase from *Brevibacterium sterolicum* related to the interconversion between open and closed channel enzyme conformations. This event regulated the oxygen diffusion and constituted the rate-limiting step preceding flavin reoxidation^29,30^. Regarding F397A and F397L, they display slower reoxidation rates probably due to the absence of an aromatic residue in that position. Moreover, both variants display slow phases that are dependent on O_2_ concentration, that could reveal two different kinetic processes, the H and H^+^ transfers recently reported to take place in separate kinetic steps in native AAO^24^.

Finally, Phe397 also plays a role in controlling the product release from the active site, with this catalytic step limiting the turnover of the F397Y and F397W variants, while the F397A and F397L variants (and native AAO) let the substrate and product diffuse easily in and out of the active site. Furthermore, ligand migration studies with both *p*-anisic acid and *p*-anisaldehyde indicate that product release is delayed due to the reduced gated space —which forces the aromatic side chains to be displaced— and the hydrogen bond between the tyrosine or tryptophan and Tyr92 in the aromatic variants. The native enzyme also requires longer times for product release because its side chain must move to let it out, although there is no hydrogen bonding between the Phe397 and Tyr92. This is in agreement with the 10-fold lower *k*_dis_ for the aromatic variants than for native AAO. Flavoenzymes with catalytic cycles limited or partially limited by the product release have been reported, such as D-amino acid oxidase^31^, cyclohexanone monooxygenase^32^ or nitroalkane oxidase^33^. The case of amadoriase I is also relevant, since its catalysis is partially limited by the product release, although the oxidation of the enzyme takes place through an ordered ternary complex of enzyme, product and O_2_^34^.

In conclusion, Phe397 plays a central role in the catalysis of *P. eryngii* AAO, which is reinforced by its high conservation among other sequences of characterised and putative AAOs from fungal genomes. As drawn from the experimental and computational results, this residue supports both half-reactions of the enzyme. During the reductive reaction, it helps the substrate attain a catalytically relevant position and facilitates product release from the active site. Analysis of the oxidative reaction suggests that the presence of an aromatic residue at this position is important to modulate the flavin environment and to reduce the space in the active site enabling the enzyme to reduce O_2_ to H_2_O_2_. Experimental and computational results complement each other well and, thus, highlight the suitability of the recently developed adaptive-PELE^22^ for the study of protein binding to different ligand types.

## Methods

### Reagents

Glucose oxidase type VII from *Aspergillus niger*, glucose, *p*-methoxybenzyl alcohol and *p*-anisic acid were purchased from Sigma-Aldrich.

### Enzyme expression and purification

Native AAO from *P. eryngii* (GenBank accession number **AF064069**) and its mutated variants F397A, F397Y, F397W and F397L were heterologously expressed in *E. coli* W3110 as recombinant proteins using the pFLAG1 vector. The above-mentioned AAO variants were prepared using the QuickChange® site-directed mutagenesis kit.

PCR reactions were run with the following oligonucleotides harbouring the desired mutations (underlined nucleotide/s in bold triplets indicated below) and the AAO gene inserted in the vector as a template: (i) F397Y: 5′-CTTTTCCAACCAATGG**TAC**CACCCAGCTATCCCTCG-3′, (ii) F397A: 5′-CTTTTCCAACCAATGG**GCC**CACCCAGCTATCCCTCG-3′, (iii) F397W: 5′-CTTTTCCAACCAATGG**TGG**CACCCAGCTATCCCTCG-3′, and (iv) F397L: 5′-CTTTTCCAACCAATGG**TTG**CACCCAGCTATCCCTCGC3′, along with their reverse complementary counterparts. The AAO variants were expressed as inclusion bodies, subsequently *in vitro* activated in the presence of FAD and purified by anion-exchange chromatography as previously described^35^.

Molar absorbance coefficients of the variants were calculated by heat denaturation of the proteins and determination of the FAD released. The coefficients for native AAO and the F397Y, F397A, F397W and F397L variants are ε_463 nm_ = 11050 M^−1^·cm^−1^ ^3^, ε_462 nm_ = 10415 M^−1^ cm^−1^, ε_459 nm_ = 9432 M^−1^·cm^−1^, ε_462 nm_ = 9952 M^−1^·cm^−1^ (at 462 nm), and ε_462 nm_ = 12492 M^−1^ cm^−1^, respectively.

### Steady-state kinetics

Bi-substrate kinetics of the four AAO variants were measured recording *p*-anisaldehyde production (Δε_285_ = 16950 M^−1^·cm^−1^), varying both the concentrations of *p*-methoxybenzyl alcohol and O_2_ in a spectrophotometer. Reactions were performed in screw-cap cuvettes, where different O_2_ concentrations in 0.1 M phosphate at pH 6.0, were attained by bubbling several O_2_/N_2_ mixtures inside a thermostatic bath at 12 °C. Reactions were triggered by the addition of the alcohol substrate (5–500 μM) and the enzyme (3–5 nM) using microsyringes. The spectrophotometer was maintained at the desired temperature owing to the use of a thermostatic bath.

Initial rates were obtained from the linear phase of the aldehyde production and were calculated as the change in absorbance over time. Kinetic constants were estimated by fitting the *k*_obs_ to equation (1) describing a ping-pong mechanism:

1 $$\frac{\nu }{{\rm{e}}}=\frac{{k}_{{\rm{cat}}}AB}{{K}_{m(\mathrm{Al})}B+{K}_{{\rm{m}}({\rm{Ox}})}A+AB}$$

where ν stands for the initial velocity, e is the enzyme concentration, *k*_cat_ is the catalytic constant, *A* stands for the alcohol concentration, *B* represents O_2_ concentration and *K*_m(Al)_ and *K*_m(ox)_ are the Michaelis constants for *p*-methoxybenzyl alcohol and O_2_, respectively.

Kinetic constants estimated as *p*-anisaldehyde and H_2_O_2_ release were compared under atmospheric O_2_ saturation conditions, at 25 °C. *p*-Anisaldehyde was measured as detailed above, while H_2_O_2_ release was estimated by coupling the reaction of a horseradish peroxidase (6 U·mL^−1^) and AmplexRed® (60 nM), which uses the H_2_O_2_ produced by AAO to give coloured resorufin (Δε_563_ = 52000 M^−1^·cm^−1^). Catalytic constants were estimated by fitting to a Michaelis-Menten equation for one substrate in both cases (equation (2)):

2 $$\frac{\nu }{{\rm{e}}}=\frac{{k}_{{\rm{cat}}}A}{{K}_{m(\mathrm{Al})}+A}\,$$

### Stopped-flow measurements

Experiments were performed using a stopped-flow spectrophotometer from Applied Photophysics Ltd. model SX17.MV.

Enzyme-monitored turnover experiments were carried out by mixing AAO (~10 µM) with exceedingly saturating *p*-methoxybenzyl alcohol concentrations (at least 10-fold the *K*_m_ for each of the variants), under air-saturated conditions, at 25 °C. Spectral evolution of the enzymes during redox turnover was recorded between 350 and 700 nm with a photodiode array (PDA) detector.

Studies on the reductive half-reaction were performed upon mixing AAO with increasing concentrations of *p*-methoxybenzyl alcohol (0.018–0.6 mM) under anaerobic conditions. The stopped-flow apparatus was made anaerobic by flushing sodium dithionite through the system, which was then rinsed out with O_2_-free buffer. All buffers, substrates and the enzyme were poured into glass tonometers that were subsequently subjected to 20–25 cycles of evacuation and argon (Ar) flushing in order to remove O_2_. To ensure anaerobiosis, glucose (10 mM) and glucose oxidase (10 U·mL^−1^) were added after some vacuum-Ar cycles. Measurements were recorded using the PDA detector, in 50 mM sodium phosphate (pH 6.0) at 12 °C. Observed rate constants (*k*_obs_) were calculated by global fitting of the spectra with Pro-K software to a single exponential equation. Those averaged *k*_obs_ at each substrate concentration were then non-linearly fitted to equation (3), describing hyperbolic substrate dependence of *k*_obs_:

3 $${k}_{{\rm{obs}}}=\frac{{k}_{{\rm{red}}}A}{{K}_{{\rm{d}}({\rm{Al}})}+A}+{k}_{{\rm{rev}}}$$

where *k*_red_ and *k*_rev_ represent the reduction rate constant at infinite substrate concentration and its reverse reaction, respectively; *A* stands for the alcohol concentration; and *K*_d_ is the dissociation constant.

The oxidative half-reaction was investigated by mixing reduced AAO with increasing O_2_ concentrations. Procedures were as explained above for the reductive half-reaction, except that, in this case, AAO and glucose were put into a tonometer bearing a side-arm, where *p*-methoxybenzyl alcohol (1.3-fold the concentration of AAO) was poured along with glucose oxidase. After the required vacuum-Ar cycles, enzyme and substrate were mixed before being mounted onto the stopped-flow equipment. Reactions were measured with both the PDA and the monochromator detectors at 12 °C. *k*_obs_ were obtained by either global fitting of the spectra or fitting the monochromator traces to exponential equations describing two-step and three-step processes. Fitting averaged *k*_obs_ either to equation (4) that describes a linear dependence on O_2_ concentration or equation (5) that describes hyperbolic saturation with increasing O_2_ concentration allowed the estimation of the apparent second-order rate constant for reoxidation (^app^*k*_ox_) and the first-order rate (*k*_ox_) and second-order constants for reoxidation (*k*_ox_/*K*_d(ox)_), respectively:

4 $${k}_{{\rm{obs}}}={}^{{\rm{app}}}k_{{\rm{ox}}}{{\rm{O}}}_{2}+{k}_{{\rm{rev}}}$$

5 $${k}_{{\rm{obs}}}=\,\frac{{k}_{{\rm{ox}}}/{K}_{d({\rm{ox}})}{{\rm{O}}}_{2}}{1\,+({k}_{{\rm{ox}}}/{K}_{d({\rm{ox}})}{{\rm{O}}}_{2})/{k}_{{\rm{ox}}}}$$

Estimation of the rates of the AAO:*p*-anisic acid complex formation and dissociation were performed by analyzing spectral changes upon mixing enzyme (~10 µM) with different concentrations of the ligand (0.04–2 mM) at 12 °C. Data were globally fitted to an equation describing a one-step process. The obtained *k*_obs_ were linearly depended on the ligand concentration and were, thus, fitted to equation (6):

6 $${k}_{{\rm{obs}}}={k}_{{\rm{for}}}{\rm{L}}+{k}_{{\rm{dis}}}$$

in which *k*_for_ stands for the second-order rate constant for the complex formation; L represents the ligand concentration, and *k*_dis_ is the rate constant for the complex dissociation.

### Spectral characterization of the AAO-*p*-anisic acid complex

The affinity of the native AAO and its variants for *p*-anisic acid was assessed by titrating the enzyme with increasing concentrations of the ligand in 50 mM sodium phosphate (pH 6.0) at 12 °C. Spectral changes were recorded using a spectrophotometer and their magnitude upon complex formation was fitted to equation (7), which accounts for a 1:1 stoichiometry, as a function of *p*-anisic acid concentration:

7 $${\rm{\Delta }}A=\frac{{\rm{\Delta }}{\rm{\varepsilon }}({\rm{E}}+{\rm{L}}+{K}_{{\rm{d}}})-{\rm{\Delta }}{\rm{\varepsilon }}\sqrt{{({\rm{E}}+{\rm{L}}+{K}_{{\rm{d}}})}^{2}-4{\rm{EL}}}}{2}$$

in which ΔA accounts for the observed change in absorbance; Δε represents the maximal absorption difference in each of the spectra; *K*_d_ is the dissociation constant; and E and L, the enzyme and *p*-anisic acid concentrations.

### Molecular dynamics (MD) studies

The protonation states of the titratable amino acids present at the AAO crystal structure (PDB 3FIM) were set up using PROPKA^36^ of *Protein Preparation Wizard*^37^ and checked with the H++ server^38^. The selected mutations (F397W, F397Y, F397A and F397L) were introduced and all 3D structures were minimized with Prime^39,40^. The overall systems were solvated and neutralized using Desmond^41^ with the SPC water model and 0.15 M of NaCl.

50 ns of MD simulation were performed with Desmond^41^ for each protein structure using the default Desmond’s protocol. The temperature was regulated with the *Nosé-Hoover* chain thermostat^42–44^ with a relaxation time of 1 ps. The pressure was controlled with the *Martyna-Tobias-Klein barostat*^45^ with isotropic coupling and a relaxation time of 2 ps. RESPA integrator^45,46^ was used with bonded, near and far time steps of 2.0, 2.0 and 6.0 ps, respectively. Furthermore, a 9 Å cut-off was employed for nonbonded interactions with the smooth particle *mesh Ewald method*^47^.

### Ligand diffusion

The minimized models described above were also used to study the ligand diffusion. The acid product was manually docked inside the cavity, next to Phe397 and Tyr92, while reactants were docked in their catalytic position. The FAD cofactor was protonated to its semiquinone state for the O_2_ simulations, while it remained in its quinone state for the acid, aldehyde and alcohol diffusion studies.

The FAD cofactor was optimized with QM/MM at the M06/6–31G* level of theory using Qsite^48^ and the atomic charges were obtained from the electrostatic potential (ESP). Ligand geometries were optimized at the same level of theory in gas phase and re-optimized using the PBF implicit solvent with the Jaguar software^49^. The ligands were parameterized according to the OPLS 2005 force field, maintaining the ESP charges, and a rotamer library was build using *Macromodel*^37^.

Ligand diffusion was modelled with the recent adaptive-PELE technique^22^, using an anisotropic network model (ANM) backbone perturbation^50^ applied to all Cα atoms, side chains re-sampling within 6 Å of the ligands, and a full energy minimization in each PELE step. The products were randomly translated and rotated within a spherical box of 22 Å of its initial position. The O_2_ and the alcohol reactants were randomly roto-translated within a spherical box of 3 Å and 7 Å of its centre of mass initial position, respectively, to inquire about their poses in the active centre. Adaptive-PELE simulations involved ~50 epochs of 4 PELE steps. All simulations were performed using 32 processors. The O_2_ simulations were executed during 72 h, while the other simulations were performed during 23 hours.

### Data availability statement

All data generated or analysed during this study are included in this published article (and its Supplementary Information files).

## Electronic supplementary material

Supplementary Information
