# Supplementary material for: Multiple implications of an active site phenylalanine in the catalysis of aryl-alcohol oxidase
Source: Sci Rep. 2018 May 25;8:8121. doi: 10.1038/s41598-018-26445-x (PMC5970180; doi:10.1038/s41598-018-26445-x)
Supplement: Supplementary file 1 — Supplementary Information [file 41598_2018_26445_MOESM1_ESM.pdf]

Multiple implications of an active site phenylalanine in the catalysis of aryl-alcohol oxidase

Juan Carro, Pep Amengual-Rigo, Ferran Sancho, Milagros Medina, Victor Guallar, Patricia Ferreira, Angel T. Martínez

Supplementary Information

Supplementary information is composed of Supplementary Table S1 and Supplementary Figures S1, S2, S3, S4, S5 and S6.

Supplementary table S1. Comparison of reaction constants measured as *p*-anisaldehyde or H_2O_2 production for Phe397 AAO variants at 25°C

	p-anisaldehyde			H_2O_2		
	k_{cat} (s^{-1})	K_m (μM)	k_{cat}/K_m ($s^{-1}·mM^{-1}$)	k_{cat} (s^{-1})	K_m (μM)	k_{cat}/K_m ($s^{-1}·mM^{-1}$)
AAO[1]	113 ± 2	30 ± 2	3770 ± 260	106 ± 5	33 ± 6	3212 ± 603
F397Y	77 ± 2	23 ± 2	3400 ± 300	71 ± 2	23 ± 2	3060 ± 310
F397W	127 ± 6	123 ± 17	1030 ± 150	124 ± 4	125 ± 11	995 ± 94
F397A	54 ± 1	35 ± 3	1560 ±140	48 ± 1	69 ± 4	698 ± 40
F397L	97 ± 6	155 ± 28	624 ± 120	92 ± 2	173 ±17	530 ± 53

Reactions performed in 50 mM sodium phosphate pH 6.0 at 25°C and at a fixed O_2 concentration of 0.26 mM. *p*-Anisaldehyde release measured as increase of absorbance ($\Delta\varepsilon_{285}$ = 16950 $M^{-1}·cm^{-1}$). H_2O_2 release measured through a coupled enzymatic reaction with horseradish peroxidase (0.5 mM) and AmplexRed® (0.35 mM) measured as increase in absorbance ($\Delta\varepsilon_{563}$ = 52000 $M^{-1}·cm^{-1}$).[1]From Ferreira et al.[22]. Means and standard deviations calculated from the fit to Michaelis-Menten equation. All kinetics were measured by triplicates.

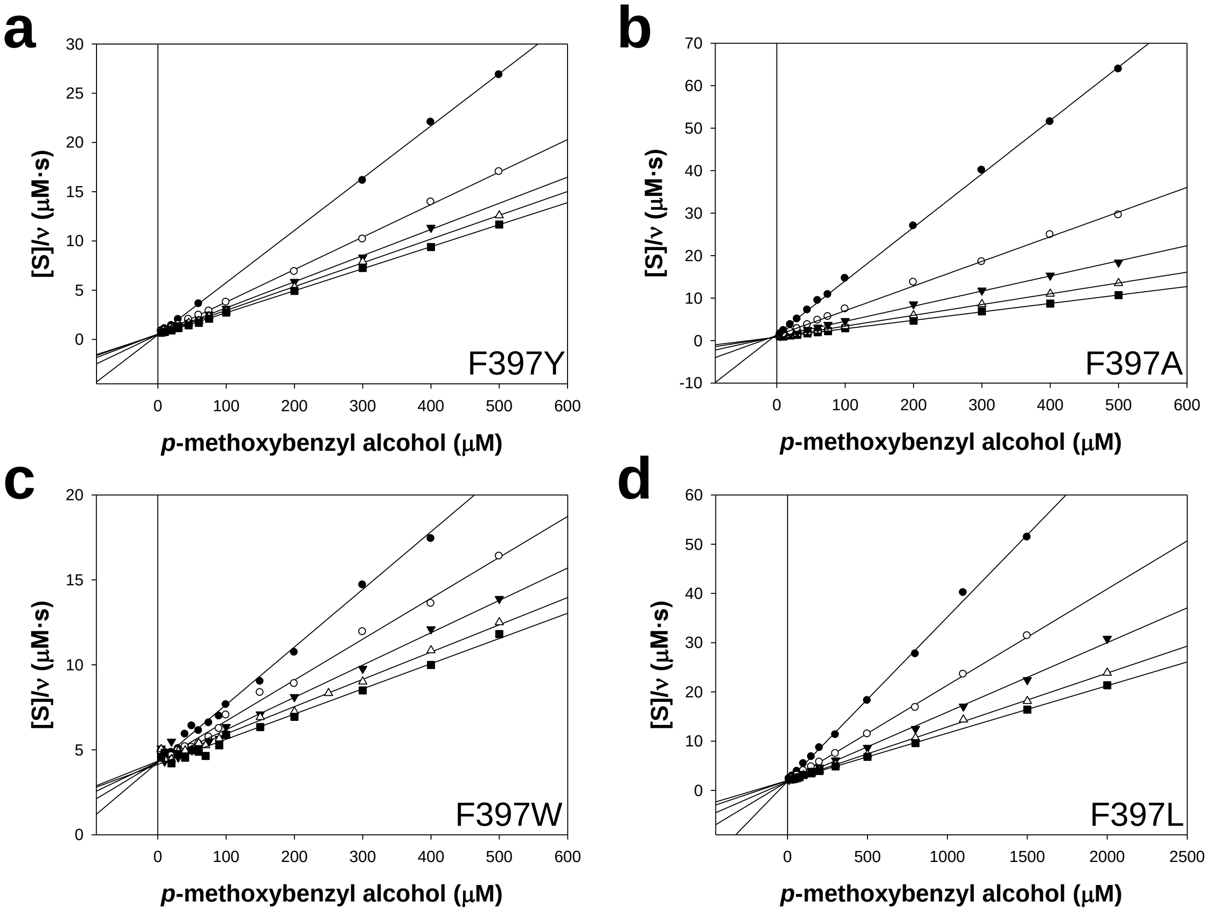

Supplementary figure S1. Linearized Hanes-Woolf plots of the bi-substrate kinetics of the Phe397 AAO variants. A. F397Y, **B.** F397A, **C.** F397W and **D.** F397L. Data were measured by varying the concentrations of *p*-methoxybenzyl alcohol and O_2 in 50 mM sodium phosphate pH 6.0 at 12°C. Vertical solid line represents x = 0. Filled circles, 0.06 mM O_2; open circles, 0.16 mM O_2; filled triangles, 0.34 mM O_2; open triangles, 0.71 mM O_2; and filled squares, 1.60 mM O_2.

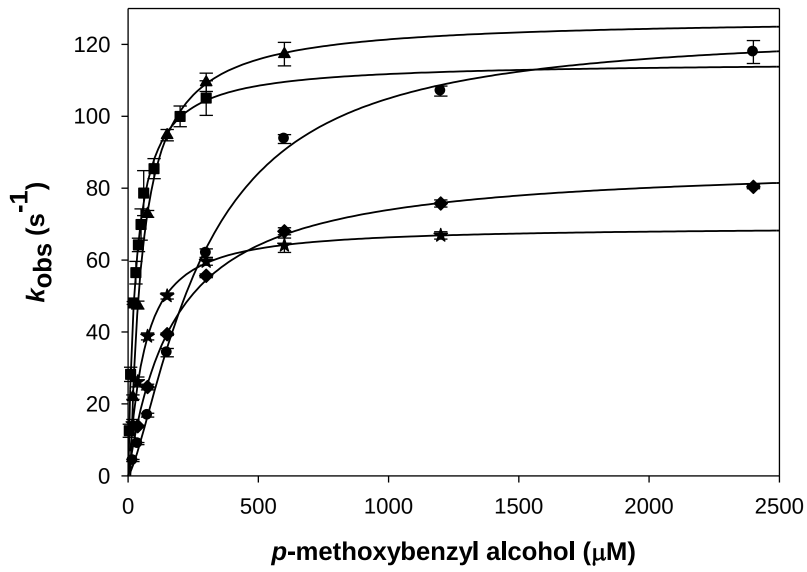

Supplementary figure S2. Plots of the observed rate constants (k_{obs}) of flavin reduction for native AAO and the four Phe397 variants. Native (squares), F397Y (triangles), F397W (circles), F397L (diamonds) and F397A (stars) AAO variants with varying concentrations of *p*-methoxybenzyl alcohol. Data were measured in an anaerobic stopped-flow spectrophotometer in 50 mM sodium phosphate pH 6.0 at 12°C. Lines represent the fits of experimental data to equation (3). Means and standard deviation calculated from triplicates.

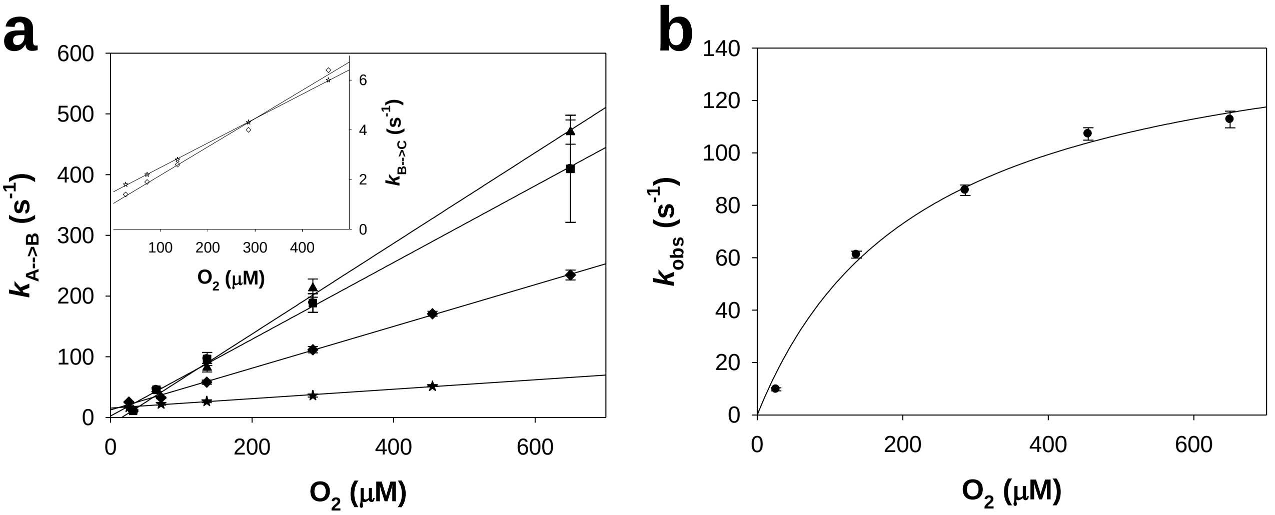

Supplementary figure S3. Plots of the observed rate constants (k_{obs}) of flavin oxidation as a function of O_2 concentration for native AAO and the four Phe397 variants. A. Native (squares), F397Y (triangles), F397A (stars) and F397L (diamonds). Inset shows the O_2-dependent k_{obs2} for the second phase of the reoxidation of F397A (open stars) and F397L (open diamonds). Lines show fit to equation (4). **B.** F397W as a function of O_2 concentration. Line shows fit to equation (5). Data were measured in a stopped-flow spectrophotometer under anaerobic conditions at 12°C and pH 6.0. Means and standard deviation estimated from triplicates.

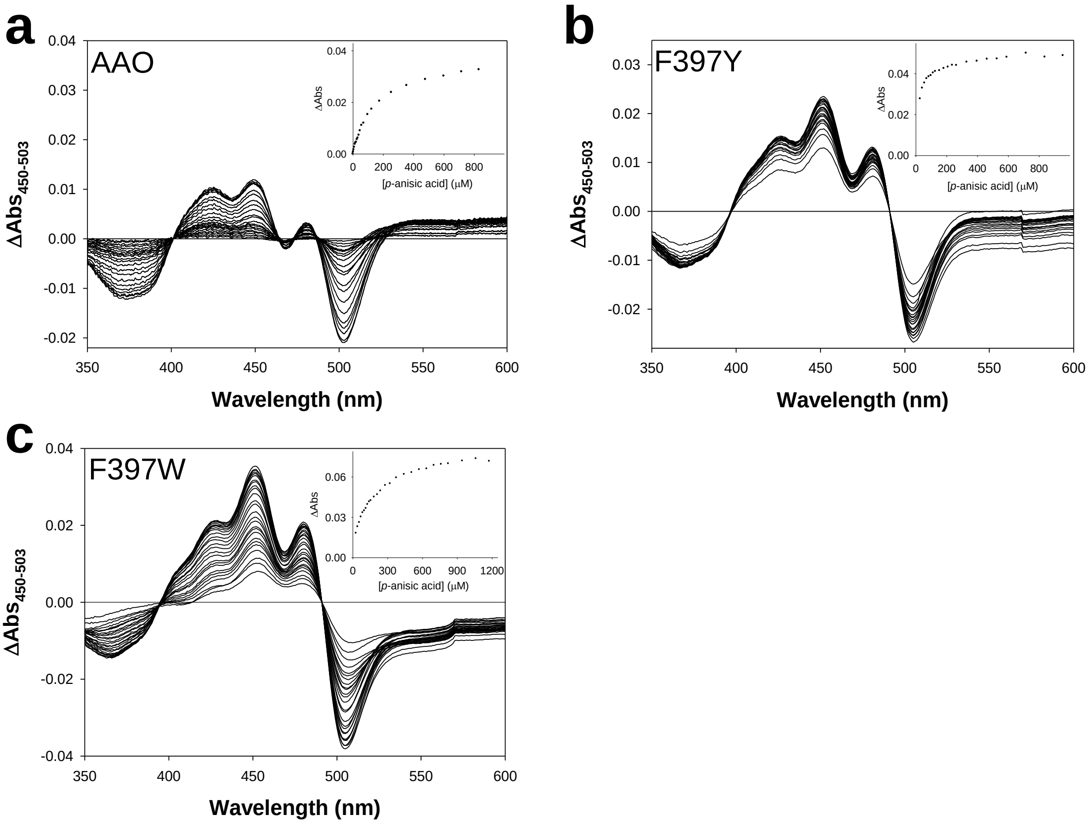

Supplementary figure S4. Spectral changes upon titration of native and Phe397 AAO variants with increasing concentrations of *p*-anisic acid. Enzyme (~10 µM, initial concentration) was titrated with *p*-anisic acid (0–1200 µM) at 25°C and pH 6.0. Spectra of the ligand-free enzyme and after each addition of ligand were recorded. All spectra were subtracted to that of the titrated enzymes to obtain the data represented. **A**. Native AAO, **B**. F397Y, and **C.** F397W variants, respectively. Insets show the differences in absorbance between maxima (450 nm) and minima (503 nm) as a function of the concentration of ligand.

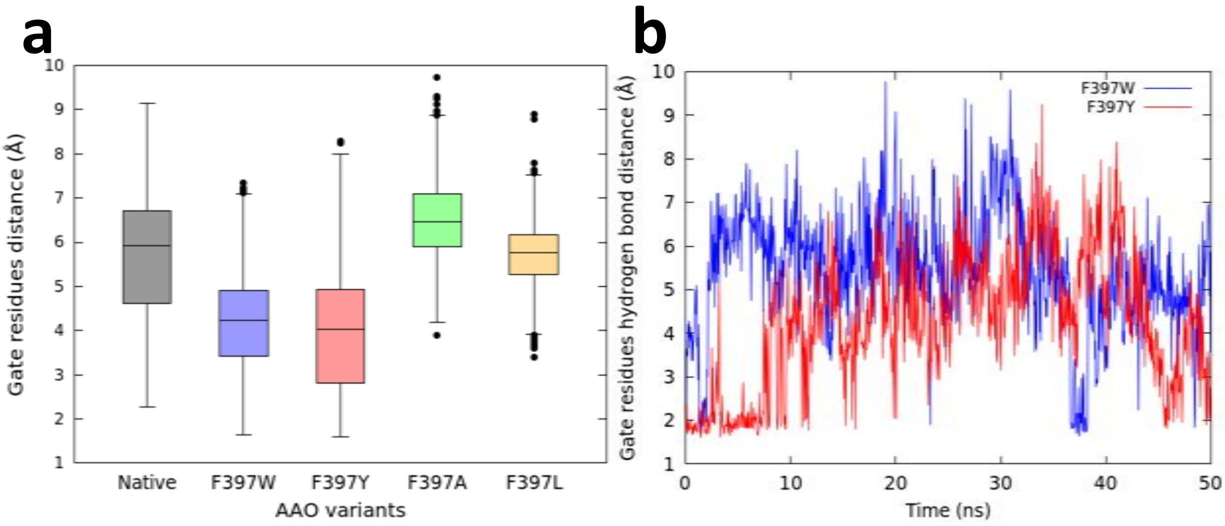

Supplementary figure S5. Distance between gate-residues (residue at 397 position and Tyr92) computed by molecular dynamics of the Gln395–Thr406 and Ser89–Met95 loops. A. Boxplots of the distances. Boxes contain 50% of the results (two quartiles), horizontal lines inside them indicate the mean value; while the upper and lower whiskers (vertical lines) contain the remaining quartiles (50%). The overall height of boxes (whiskers included) is indicative of the spread of the results. Isolated points represent results significantly different from the rest of data values. Grey, native AAO; blue, F397W; red, F397Y; yellow, F397A; and light green, F397L variants. **B.** Representation of the evolution of the distances as a function of time (0–50 ns) for F397W and F397Y variants.

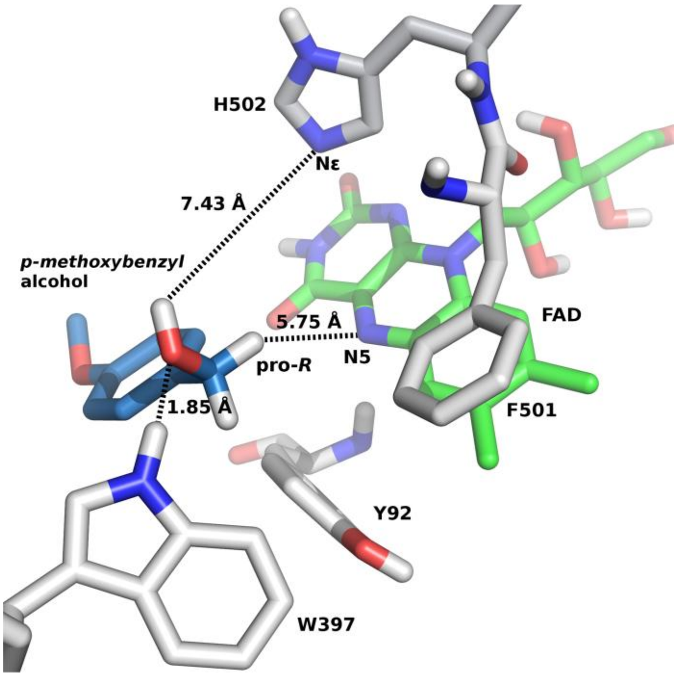

Supplementary figure S6. Representation of the energetically-favourable inefficient catalytic pose of *p*-methoxybenzyl alcohol in the F397W variant. Distances to His502, Trp397 and N5 of the FAD are indicated. Sticks in CPK, carbons in light blue.
